# Myofibroblasts reside in the middle dermis of the keloids but do not predict the response to injection therapies: a double-blinded, randomized, controlled trial

**DOI:** 10.3389/fmed.2024.1293028

**Published:** 2024-03-01

**Authors:** Tuomas Komulainen, Patrik Daymond, Kristiina E. Hietanen, Ilkka S. Kaartinen, Tero A. H. Järvinen

**Affiliations:** ^1^Faculty of Medicine and Health Technology, Tampere University, Tampere, Finland; ^2^Department of Musculoskeletal Surgery and Diseases, Tampere University Hospital, Tampere, Finland; ^3^Department of Plastic Surgery, Hospital Nova, Wellbeing Services County of Central Finland, Jyväskylä, Finland

**Keywords:** keloid, myofibroblast, fibroblast, human, double-blinded randomized controlled trial, injection, scar, fibrosis

## Abstract

**Introduction:**

Keloids form as a pathological response to skin wound healing, and their etiopathology is poorly understood. Myofibroblasts, which are cells transformed from normal fibroblasts, are believed to contribute to pathological scar formation in wounds.

**Methods:**

We carried out a double-blinded randomized controlled trial (RCT) comparing the efficacy of intralesional 5-fluorouracil (5-FU) and triamcinolone (TAC) injections in treating keloids. A total of 43 patients with 50 keloids were treated with either intralesional TAC or 5-FU injections, and their clinical response was evaluated. Biopsies were collected before, during, and after injection therapy from the active border of a keloid. To understand the role of myofibroblasts in keloids, we conducted an immunohistochemical analysis to identify myofibroblasts [α-smooth muscle actin (αSMA)] from the biopsies. We first defined the three histologically distinct regions-superficial, middle, and deep dermis-in each keloid.

**Results:**

We then demonstrated that myofibroblasts almost exclusively exist in the middle dermis of the keloids as 80% of the cells in the middle dermis were αSMA positive. However, both the percentage of myofibroblasts as well as the area covered by them was substantially lower in the superficial and deep dermis than in the middle dermis of the keloids. Myofibroblasts do not predict the clinical response to intralesional injection therapies. There is no difference in the myofibroblast numbers in keloids or in the induced change in myofibroblasts between the responders and non-responders after treatment.

**Discussion:**

This study demonstrates that myofibroblasts reside almost exclusively in the middle dermis layer of the keloids, but their numbers do not predict the clinical response to intralesional injection therapies in the RCT.

## Introduction

Keloids represent a dermal fibrotic disorder that occurs following an aberrant wound-healing response, which leads to excessive scar formation. Keloid scars are marked by the excessive accumulation of extracellular matrix (ECM) in the skin (1–3). There are therapeutic challenges in the treatment of keloids. Moreover, there are currently no keloid-specific treatments available that can make the keloids disappear from the affected anatomical area. Although a large number of therapeutic treatments have been tested and used to treat keloids, their response rates are low but recurrence rates remain high. This is partially due to a poor understanding of keloid biology and its pathogenesis. A more thorough understanding of keloid biology could potentially lead to improved therapeutics, which, in turn, can be used in the treatment of keloids (4–7).

Myofibroblasts are specialized cells transformed from normal fibroblasts under the influence of transforming growth factor-β (TGF-β) and the mechanical strain placed upon them (8–15). They are contraction-capable cells that produce and organize ECM into scar tissues. Scar tissues effectively and quickly restore the mechanical integrity of lost tissue architecture but cause the loss of tissue functionality (8–13). Due to their role in scar formation, myofibroblasts are theorized to play a role in pathological scar formation in skin wounds (16, 17). However, their exact role in keloid pathogenesis has remained elusive (11). It has been recently demonstrated that myofibroblasts are responsible for excessive production of collagen in keloids (18, 19). On the other hand, it is not entirely clear whether keloids represent a “myofibroblast disease,” i.e., whether myofibroblasts contribute to keloid formation (20, 21). To explore their potential role in keloid biology, we studied myofibroblasts using biopsies collected from 43 patients with 50 keloids requiring treatment. All the patients underwent a double-blinded randomized controlled trial (RCT) comparing two commonly used intralesional therapeutic drugs: triamcinolone acetate (corticosteroid; TAC) and 5-fluorouracile (5-FU) (22). We collected histological biopsies from the keloids before, during, and after the therapeutic injections and stained the histological sections for myofibroblasts to explore the role of myofibroblasts in keloids with a special emphasis on three recently described histologically distinct layers that exist in keloids (22, 23). We demonstrate that the myofibroblasts reside almost exclusively in the middle dermis layer of the keloid. However, myofibroblasts do not predict the clinical response to the injection therapies. The change induced in the myofibroblast population is not explanatory for the clinical response to injection treatments in human keloids.

## Materials and methods

### Patients

The study was approved by the ethics committee of the Pirkanmaa Health Care District and recorded in the prospective clinical studies database: ClinicalTrials.gov (#NCT02155439).

A total of 43 patients with 50 active and symptomatic keloids requiring treatment were enrolled and randomized into two groups (Supplementary Figures 1, 2) (22). The keloids were randomized to either 5-FU- or TAC-treated groups with a permutated-block randomization. The treated keloids were categorized as responders and non-responders at the end of the 6 months of follow-up. The group characteristics are given in Supplementary Table 1. The remission of the keloid can be clinically defined by an experienced plastic surgeon as the flattening of the keloid to such a degree that no further treatment or injections are indicated. The collection of clinical data was blinded to the observing plastic surgeon who was not aware of the treatment group assignment (22).

A keloid was clinically defined as a tumor-like lesion growing outside the boundaries of the original wound site. Surgical wounds were also considered as keloids if the scar has not shown signs of resolution for over 3 years. The etiologies and anatomic locations of the keloids have been described in detail in our previous publication, where the clinical outcomes of the RCT were reported (22).

### Double-blinded randomized controlled trial (RCT)

The patients were treated with intralesional injections of either TAC or 5-FU at 3- to 4-week intervals. All the patients visited the outpatient clinic a total of five times (once every 3–4 weeks until week 12 and at 6 months). The injections were given by the same experienced plastic surgeon (IK) according to the international recommendations. For patients who did not need three injections, control visits were carried out (Supplementary Figure 2) (22).

For TAC injections, 20 mg/ml of Lederpan^®^ (Haupt Pharma Wolfratshausen GmbH, Germany) mixed at a ratio of 1:1 with 10 mg/ml of lidocaine (Orion Pharma, Finland) was used. For 5-FU injections, 5-Fluorouracil Accord (AccordHealthCare Ltd North Harrow, UK) was used at a concentration of 50 mg/ml.

During the first three visits, a 3-mm punch biopsy was obtained from the active border of the keloid for histological and immunohistochemical (IHC) analyses. The first biopsy was obtained before any treatment. The second and third biopsies were obtained after the first and second injections at 4 and 8 weeks, respectively. The third biopsy was not conducted if the treatment was stopped because of the favorable reaction of the keloid (22).

### Histopathology

Punch biopsies from the keloids were fixed with 4% paraformaldehyde and processed according to standard histological methods. The tissue microarray (TMA) technique was applied to the biopsies. In brief, each punch biopsy was split in a sagittal direction, and then, the biopsies were assembled into a TMA paraffin block using TMA Master II, a computer-controlled machine that places the biopsies in an orderly fashion. Each histological TMA block encompassed all the biopsies (1–3) from five consecutive patients, resulting in 10 TMA blocks (+ 10 identical replicas) with 10–15 punches. One routine hematoxylin–eosin (HE) staining was performed to analyze the basic histological characteristics and to confirm the representativeness of each biopsy (24). A board-qualified pathologist examined all the specimens and determined specimen adequacy (22). Each keloid was classified into three distinct regions—superficial, middle and deep dermis—according to the new histological evaluation system (23). The investigators were blinded to all examinations and analyses.

### Immunohistochemistry (IHC)

The IHC analysis was performed on 6-μm thick paraffin sections, as previously described in detail elsewhere (10, 15, 25). For the analysis, the following primary antibodies were used: M0851 1A4 mouse anti-human α-smooth muscle actin (αSMA; DakoCytomation, Glostrup, Denmark) and M0823 mouse anti-human cluster of differentiation 31 (CD31, DakoCytomation, Glostrup, Denmark), followed by the appropriate horseradish peroxidase-conjugated secondary antibodies (Immonlogic anti-mouse IgG DPV55HRP, Duiven, Netherlands) (11). The blocking reagents used for IHC were S2O23 REAL and S0809 Antibody Diluent (DakoCytomation). The peroxidase-reactive chromogen used was diaminobenzidine (K3465, DAKO, Agilent Technologies).

### Virtual microscopy and quantitative IHC analyses

IHC-stained histological sections were scanned to produce digital images using an Olympus^®^ VS200 Slideview research scanner. Image analysis and the quantification of IHC parameters were performed using open-source pathology image analysis software QuPath version 0.4.3 (26). The regions-of-interest (ROIs) were drawn manually for each keloid part (superior, middle and deep dermis) according to Jiao et al. (23).

The percentage of αSMA- and CD31-positive pixels, the percentage and density αSMA-positive cells, and the overall cell density in each keloid part were measured. The thresholds defining positivity and negativity for αSMA- and CD31-positive pixels and αSMA-positive cells were selected manually based on manual estimations to distinguish real positive pixels or cells from background staining, and the same threshold regarding pixels or cells was applied to all ROIs in all the samples. Each pixel or cell was classified as either positive or negative based on the intensity of the αSMA- or CD31-staining.

### Statistical analysis

Statistical analyses were performed with GraphPad Prism version 9.0.0 for Windows (GraphPad Software, San Diego, CA; www.graphpad.com). The normality of the distribution of data was analyzed with D'Agostino–Pearson normality tests and histograms. A repeated measures mixed-effects model with or without the assumption of equal sphericity with or without Geisser–Greenhouse correction followed by the Bonferroni multiple comparisons test was used to compare the quantities between the three different sits of the keloid dermis. Depending on data distribution, an unpaired two-tailed *t*-test with or without Welch's correction or the Mann–Whitney test was used to test if the difference between the responder and the non-responder groups before or after treatment or if the difference in certain group characteristics was statistically significant. The chi-square test was employed to analyze differences in group characteristics if the variable was categorical. A paired two-tailed *t*-test or Wilcoxon matched pairs signed rank test was used to analyze the statistical significance of the change in quantities during treatment in each part of the keloid in both responder and non-responder groups. If the same data were used in multiple comparisons, the Bonferroni correction was applied to the results by multiplying it with the *P*-value. Spearman's correlation coefficient was used to analyze the covariation between the lesion sizes measured in area (mm^2^) and percentage of αSMA-positive cells. A *P*-value of < 0.05 was considered statistically significant.

## Results

### Myofibroblasts reside in the middle dermis of keloid

The clinical results of the RCT comparing intralesional injection of TAC to 5-FU have been published. The clinical outcome was not statistically significantly different between the groups receiving either TAC or 5-FU (22). The remission rate was 46% for the 5-FU group and 60% for the TAC group at 6 months (*non-significant*) (22). For the current study, the treatment groups were combined, and the patients were classified either as responders or non-responders (Supplementary Figures 1, 2). The clinical characteristics of the non-responder and responder groups are illustrated in Supplementary Table 1. The non-responder and responder groups did not differ significantly.

Recently, a new histological evaluation system that identifies three distinct regions—superficial, middle and deep dermis—in keloids has been introduced (23). We next identified these three distinct regions in keloids from both HE- and αSMA-stained tissue sections to assess the prevalence of myofibroblasts in different layers of keloids. The myofibroblasts were determined by the IHC staining of αSMA, which is widely used as a marker of myofibroblasts (19). The size of the middle dermis was substantially larger in size than the superficial (4.7-fold) and the deep dermis (3.4-fold; middle-superficial *P* < 0.0001, middle-deep *P* < 0.0001, superficial-deep *P* = 0.0059) in the biopsies, comprising almost 66.3% of the biopsies on average (Supplementary Table 2). Cell density was similar between the superficial and middle dermis (1,465/mm^2^ and 1,589/mm^2^) but significantly higher in the deep dermis (2,188/mm^2^) before treatment (Supplementary Table 2).

**Figure 1:**
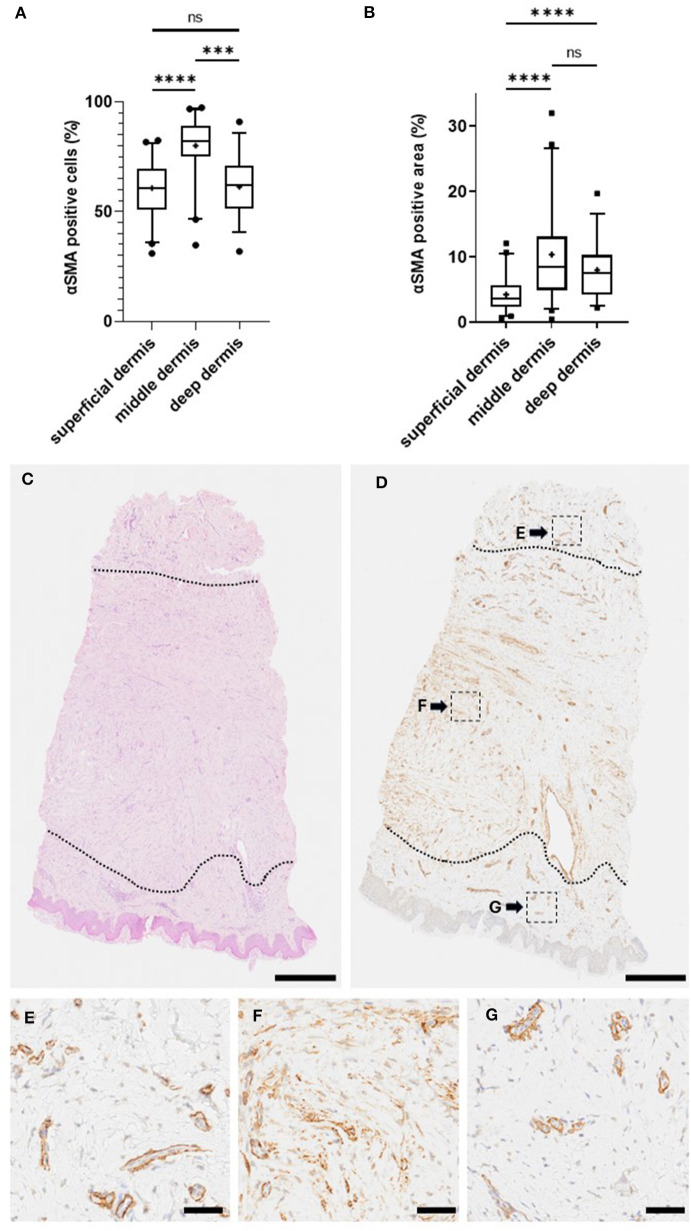
Myofibroblasts reside in the middle dermis of keloid. Biopsies were collected from the active border of 50 human keloids. The histological sections were stained for myofibroblasts (using antibodies against αSMA), while adjacent tissue sections were stained with an endothelial cell marker (antibodies against CD31). **(A)** Percentage of myofibroblasts in different regions of keloids. Most of the myofibroblasts reside in the middle dermis of the keloid. ****P < 0.0001, ***P < 0.001, ns = *P* > 0.05. **(B)** Blood vessel-positive area was reduced from the αSMA-positive area to obtain the area covered by myofibroblasts in different regions of keloids. The analysis confirms the highest proportion of myofibroblasts residing in the middle dermis of the keloid. **(C–G)** Representative histological images of myofibroblasts in different regions of keloid (Olympus VS200 Slideview, ORCA-Fusion C14440 Hamamatsu, Olympus UPlanXApo 20 × /0.80). **(C)** HE-stained biopsy from human keloid, where different keloid regions have been identified (dotted line). **(D)** The same biopsy stained for αSMA. **(E–G)** High magnification images of αSMA staining from different regions of keloid. **(D)** Superficial **(G)**, middle **(F)**, and deep **(E)** dermis. Bars: **(C**, **D)** 500 μm, **(E–G)** 50 μm.

Once the ROIs had been determined, we utilized the automated image analysis software to determine the percentage of myofibroblasts (percentage of positive cells and area (= pixels) that were stained with αSMA) in the untreated keloids. Our analysis demonstrated that the proportion of myofibroblasts is significantly higher in the middle dermis than either in the superficial or deep dermis parts of the keloids. The mean percentage of cells that stained positively for αSMA before the treatment was higher in the middle dermis (80.0%) than the deep (61.2%) and superficial (60.7%) dermis (middle vs. deep *P* < 0.0001, middle vs. superficial *P* < 0.0001, superficial vs. deep, *P* > 0.9999; Figure 1). This phenomenon was also clearly detectable under histological evaluation. The density of myofibroblasts before the treatment was as dense in the middle dermis (1,305 cells per mm^2^) as in the more cellular deep dermis (1,367 cells per mm^2^, NS) and significantly higher than in the superficial dermis (870 cells per mm^2^, *P* < 0.001, superficial vs. deep, *P* < 0.0001).

**Figure 2:**
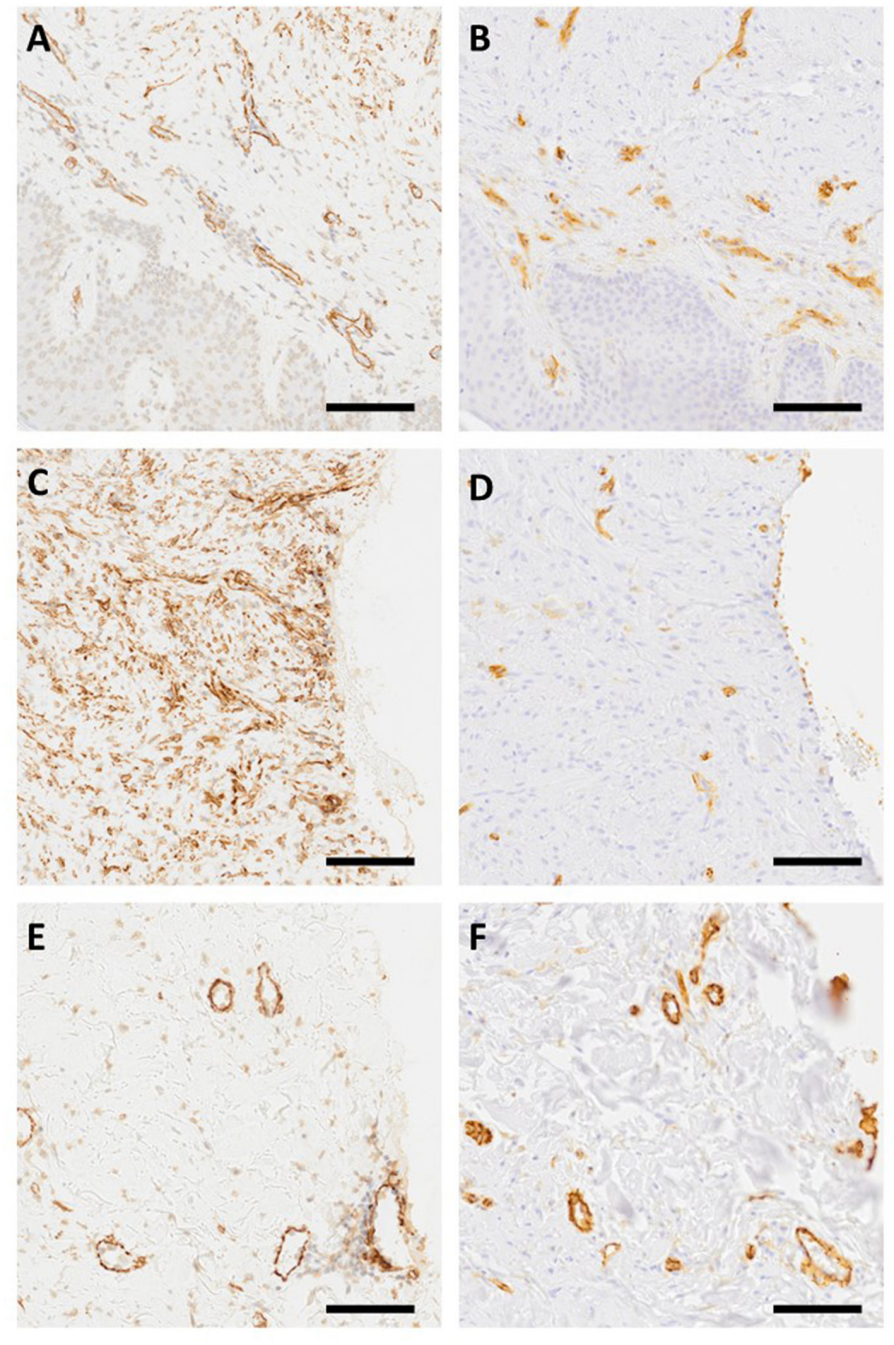
Myofibroblasts and blood vessels in keloids. Adjacent tissue sections from keloids were stained with αSMA **(A, C, E)** and endothelial cell marker CD31 **(B, D, E)** antibodies (Olympus VS200 Slideview, ORCA-Fusion C14440 Hamamatsu, Olympus UPlanXApo 20 × /0.80). The figures demonstrate that myofibroblasts are not the only αSMA-positive cells in keloids as there are αSMA-positive cells in blood vessels. Representative images from superficial **(A, B)**, middle **(C, D)**, and deep **(E, F)** dermis stained with αSMA and CD31 antibodies. Bar 100 μm.

As the microscopical evaluation illustrated that a fraction of the αSMA-positive cells is from the blood vessels (Figure 2), i.e., smooth muscle cells expressing αSMA, we stained the adjacent tissue sections from each keloid with an endothelial cell marker (CD31) to visualize the blood vessels (Figure 2). After staining, we subtracted the area staining positively for CD31 from the αSMA-positive area to obtain vasculature-free αSMA-positive staining, i.e., a score that would represent myofibroblasts more specifically. The percentage of area that stained positive for αSMA after the “blood vessel subtraction” was greater in the middle dermis (10.3%) than in the deep dermis (7.95%; *P* = 0.0893) or in the superficial dermis (4.20%; *P* < 0.0001, superficial vs. deep *P* < 0.0001; Figure 1). Taking into account the size of the middle dermis in keloids, 68.9% of the keloid myofibroblasts reside in this region in the biopsies. The “vasculature-free” area was used in the further analyses to represent the area occupied by myofibroblasts.

### Myofibroblasts do not correlate with the keloid size

The size of the keloid lesions was measured at the start of the RCT. Since clinically larger keloids are known to have a higher rate of recurrence, we analyzed the association between the keloid size and the fraction of myofibroblasts in each dermal layer of the biopsies taken from the active border of the keloid (27). We observed no correlation between the keloid size (mm^2^) and the myofibroblast percentage in any of the dermal layers (superficial dermis *r* = 0.05, middle *r* = −0.05 and deep *r* = −0.03).

### Myofibroblasts do not predict the clinical response to intralesional injection therapies in the double-blinded RCT in keloid patients

**Figure 3:**
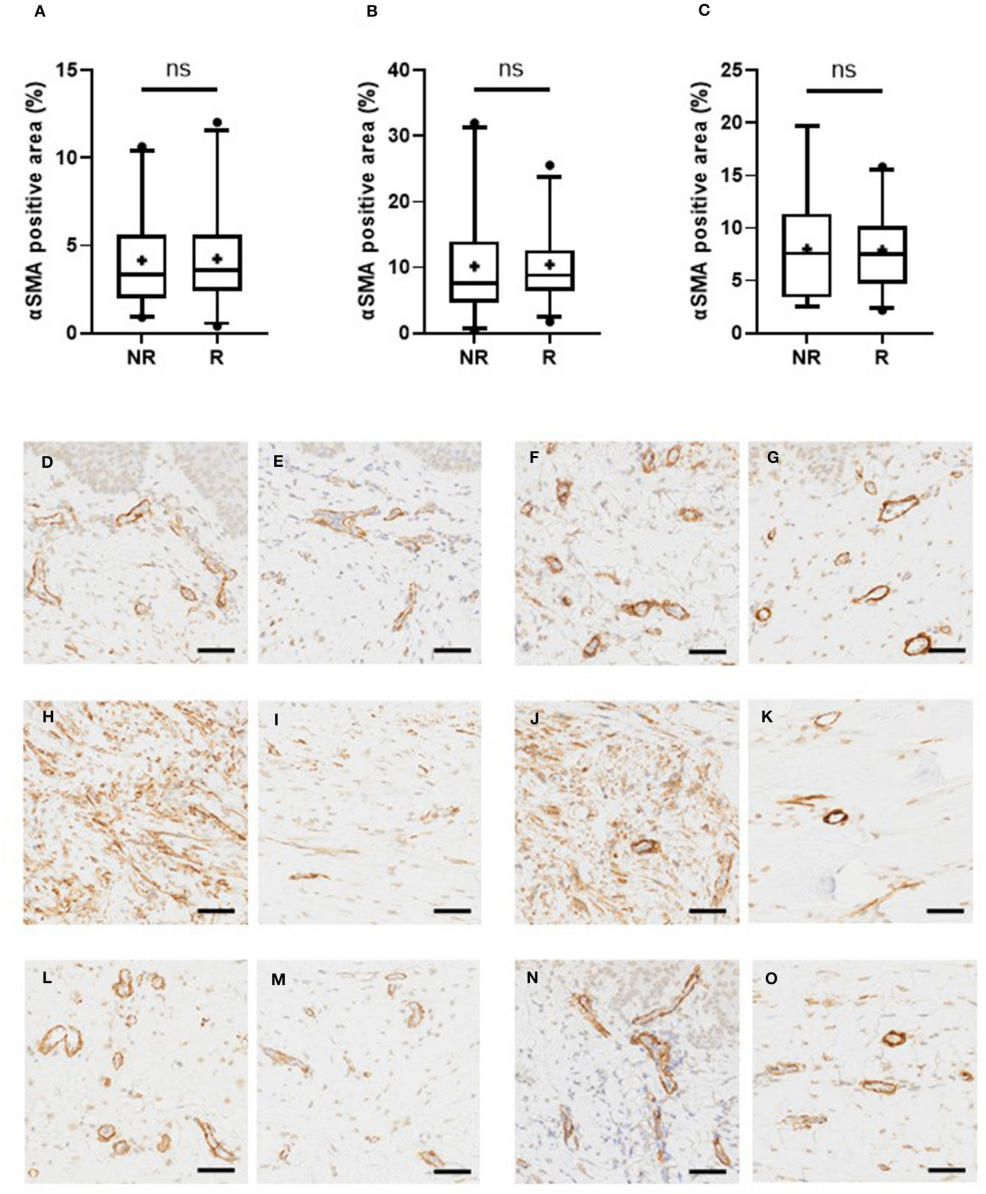
Myofibroblast-staining does not predict the response to injection therapies in keloids. The keloid patients were randomized to receive either intralesional TAC or 5-FU-injections and their clinical response was evaluated. The patients were classified either as non-responders (NR) and responders (R) according to the clinical response to injection therapies. Myofibroblast-staining was performed from the pre-treatment biopsies obtained from the active border of keloids, as described in the legend for Figure 1B. **(A–C)** Proportion of myofibroblasts in different regions of keloid, ns = *P* > 0.05. The non-responders (NR) and the responders (R) have a similar proportion of myofibroblasts in the superficial **(A)**, middle **(B)**, and deep **(C)** dermis of the keloid. **(D–O)** Representative histological images from the keloids (Olympus VS200 Slideview, ORCA-Fusion C14440 Hamamatsu, Olympus UPlanXApo 20 × /0.80). Rows presenting different parts of keloid dermis (top down superficial, middle, and deep dermis) and columns presenting different patients. Patients not obtaining clinical response to intralesional injection therapy are in the first two columns **(D, H, L and E, I, M)** and obtaining the response in the last two **(F, J, N and G, K, O)**. The keloids **(D, H, L)** and **(F, J, N)** have high expression of αSMA, while keloids **(E, I, M)** and **(G, K, O)** have low αSMA expression despite opposite clinical responses. Bar: 50 μm.

As we utilized the biopsies collected from the double-blinded RCT comparing intralesional injection therapies, we next wanted to explore whether the myofibroblast population in different regions of keloids predicts the response to subsequent injection therapy. In the pre-treatment biopsies, no difference in the area (%) occupied by myofibroblasts was observed between the non-responders (NR) and the responders (R) in the superficial (mean 4.2% vs. 4.2% *P* > 0.9999), the middle (mean 10.2% vs. 10.5 % *P* > 0.9999), or the deep (mean 8.0% vs. 7.9% *P* > 0.9999) dermis before the treatment (Table 1, Figure 3). The same result was obtained when the percentage of positive cells (Table 2) or the cell density of myofibroblasts (Supplementary Table 3) was used as the outcome measure.

**Table 1:** Area covered by myofibroblasts in keloids.

	**No. (%)**		***P*-value**
**Dermis part**	**Non- responders**	**Responders**	
**Superficial dermis**			
Mean	4.16	4.24	>0.9999
SD; range (5%−95%)	2.51; 0.93–10.39	2.69; 0.57–11.61	
**Middle dermis**			
Mean	10.19	10.46	>0.9999
SD; range (5%−95%)	8.10; 0.72–31.27	5.48; 2.54–23.73	
**Deep dermis**			
Mean	8.01	7.91	>0.9999
SD; range (5%−95%)	5.25; 2.54–19.69	3.69; 2.33–15.54	

**Table 2:** Myofibroblasts in keloids.

**Dermis part**	**No. (%)**		***P*-value**
	**Non- responders**	**Responders**	
**Superficial dermis**			
Mean	59.12	62.09	>0.9999
SD; range (5%−95%)	14.96; 31.83–82.00	11.22; 34.35–83.54	
**Middle dermis**			
Mean	77.16	82.55	0.9026
SD; range (5%−95%)	15.68; 37.04–94.61	11.86; 51.98 – 97.14	
**Deep dermis**			
Mean	57.32	63.75	0.6326
SD; range (5%−95%)	13.91; 31.89–84.91	12.46; 43.12–89.75	

Finally, as the biopsies were collected before, during, and after treatment and the clinical treatment outcome was recorded in a prospective manner, we explored whether the myofibroblast population responds to intralesional injection therapies differently in both non-responders and responders. There was no statistically significant change in the area (%) occupied by myofibroblasts during the treatment in either non-responders or responders (Supplementary Table 4). The change in the positive cell proportions yielded the same outcome (Supplementary Table 5). The myofibroblast cell density increased significantly in the superficial dermis during the treatment, but in a similar fashion, in both (responder and non-responder) groups. Otherwise, no significant differences were detected in myofibroblast cell densities in different regions of the keloid (Supplementary Table 6).

There was no difference between the non-responders and responders in the myofibroblast numbers in keloids after treatment. The area covered by myofibroblasts after treatment did not differ between the non-responders and responders in the superficial (6.0% vs. 5.8%, *P* > 0.9999), middle (12.5% vs. 10.6%, *P* > 0.9999) or deep (7.1% vs. 6.9%, *P* > 0.9999) dermis. The same outcome could be observed in the fraction of myofibroblasts and myofibroblast density (Supplementary Table 7).

## Discussion

The keloids pose a therapeutic challenge to the clinicians. A more thorough understanding of keloid biology could potentially lead to improved therapeutic strategies to treat them. One such strategy could be to identify the biological marker for biopsy, which could be used as a predictive factor for any given therapy. Thus, we explored the role of myofibroblasts in keloids using biopsies collected in a double-blinded RCT comparing the most commonly used intralesional injection therapies, TAC and 5-FU (22). These biopsies collected from the active border of the keloids demonstrate that myofibroblasts reside almost exclusively in the middle dermis layer of the keloids, but their numbers do not predict the clinical response to injection therapies. There is no difference in the change induced in myofibroblasts by the treatment therapies in relation to the clinical response to intralesional injection therapies.

Myofibroblasts are contraction-capable fibroblasts that are required for scar formation during normal wound healing. They are believed to play a role in pathological wound healing conditions as well, such as hypertrophic scars, but their actual role in keloids has remained elusive and undefined. It is still debated whether keloids represent a “myofibroblast disease” as these skin lesions are characterized by the excessive accumulation of ECM rather than contractures, and contrasting results on the actual involvement of myofibroblasts in keloids have been reported (13). Some studies have even suggested that myofibroblasts are not present in keloids (20, 21), but there are also studies demonstrating that myofibroblasts are responsible for excessive collagen production in keloids (18, 19).

Recently, the single-cell RNA sequencing (RNA-seq) studies have significantly contributed to our understanding of keloid biology (28–35). Single-cell RNA-seq studies have demonstrated that there is an increase in endothelial and fibroblast populations and that a macrophage-centered communication regulatory network exists in keloids (31, 32, 35). Furthermore, these studies have shown that fibroblast heterogeneity in keloids consisting of five major fibroblast clusters and four functional fibroblast subsets (secretory-papillary, secretory-reticular, mesenchymal, and pro-inflammatory fibroblasts) have been mapped in keloids (29, 36). The mesenchymal fibroblast population is responsible for excessive collagen production. As much as 54% of the mesenchymal fibroblast population could be classified as myofibroblasts in keloids, while myofibroblasts also exist (but in substantially lower proportion) in other fibroblast populations in keloids (29). The fundamental problem of single-cell RNA-seq studies is that the morphology of the tissue is distorted by obtaining single-cell suspension. Taking into account three histologically distinct regions in keloids as well as the potential contribution of the overlying epidermis to the keloid formation (37, 38), it is of utmost importance to carry out the spatial transcriptomics studies to define the cell and gene expression profiles according to these regions in keloids. In line with this thinking, we demonstrate in this study that myofibroblasts reside almost exclusively in the middle dermis layer of keloids.

Despite the high variability in the number of myofibroblasts between different keloids, their numbers do not predict the clinical response to intralesional injection therapies in the double-blinded RCT. We acknowledge that the drug chosen for treatment strategies used in the RCT neither acts on myofibroblasts nor influences the molecular pathways involved in myofibroblast transformation. However, the difference in the clinical outcomes of intralesional injection therapies between the responders and the non-responders are substantial. Therefore, we anticipated that either drug could exert its effects on keloids through mechanisms that are dependent on myofibroblast population or/and ultimately lead to measurable changes in the keloid myofibroblasts if they are important for keloid biology. Neither of the postulated scenarios occurred as there was no association between the myofibroblasts and the clinical response to injection therapies between the responders and the non-responders. This finding leads to the possibility that other biological factors influence the outcome. Xia et al. compared the transcriptomic profiles of keloids treated with a combination of TAC and 5-FU to those of untreated keloids and normal skin (33). The authors noticed that TAC + 5-FU interrupted the differentiation of fibroblasts toward pro-fibrotic subtypes and also reduced the myofibroblast differentiation toward mesenchymal fibroblasts. These biological changes could explain the response to treatment without the need for a significant change in the myofibroblast numbers. Xia et al. (33) also observed a reduced communication between T cells and fibroblasts in the TAC + 5-FU -treated keloids, which could also account for the treatment response without significant changes in the myofibroblast numbers.

One of the strengths of the current study is its study design—a double-blinded RCT design. The biopsy samples were collected from the active border of the keloids before, during, and after treatment, while the assessment of the clinical outcome to intralesional injections was performed by the same protocol and by the same plastic surgeon in each patient. This study design allowed us to address whether myofibroblasts can be used as a predictive marker of response and also provided an opportunity to understand the biological changes that determine whether the keloids respond to injection therapy or not.

Our study demonstrates that myofibroblasts reside mainly in the middle dermis layer of the keloids, but the myofibroblast population do not predict the clinical response to intralesional injection therapies. There is no difference between the responder and non-responder groups in terms of myofibroblast numbers or in the change induced in myofibroblasts in keloids after the treatment.

## Data availability statement

The original contributions presented in the study are included in the article/Supplementary material, further inquiries can be directed to the corresponding author.

## Ethics statement

The studies involving humans were approved by Ethics Committee of the Pirkanmaa Health Care District. The studies were conducted in accordance with the local legislation and institutional requirements. The participants provided their written informed consent to participate in this study.

## Author contributions

TK: Conceptualization, Data curation, Formal analysis, Investigation, Methodology, Visualization, Writing – original draft, Writing – review & editing. PD: Investigation, Writing – original draft, Writing – review & editing. KH: Formal analysis, Investigation, Writing – review & editing. IK: Conceptualization, Formal analysis, Funding acquisition, Investigation, Project administration, Resources, Supervision, Writing – review & editing. TJ: Conceptualization, Formal analysis, Funding acquisition, Project administration, Resources, Supervision, Validation, Writing – original draft, Writing – review & editing.
