# Supplementary material for: Myofibroblasts reside in the middle dermis of the keloids but do not predict the response to injection therapies: a double-blinded, randomized, controlled trial
Source: Front Med (Lausanne). 2024 Mar 1;11:1293028. doi: 10.3389/fmed.2024.1293028 (PMC10943694; doi:10.3389/fmed.2024.1293028)
Supplement: Supplementary file 1 [file Data_Sheet_1.docx]

Supplementary Material

**Supplementary Table 1.** Non-responder and responder group clinical characteristics.

| **Characteristic** |  | **Non-responders** | **Responders** | **P** |
| --- | --- | --- | --- | --- |
| **Age, years** | ***Mean; 95 % CI*** | 41; 35–47 | 46; 39–53 | 0.3008* |
|  | ***Range*** | 20–81 | 18–81 |  |
| **Sex, n (%)** | ***Male*** | 15 (65 %) | 11 (42 %) | 0.1879** |
|  | ***Female*** | 8 (35 %) | 15 (58 %) |  |
| **Anatomic Location, n (%)** | ***Chest*** | 9 (39 %) | 7 (27 %) | 0.3162** |
|  | ***Shoulder*** | 3 (13 %) | 10 (38 %) |  |
|  | ***Upper back*** | 6 (26 %) | 4 (15 %) |  |
|  | ***Abdomen*** | 3 (13 %) | 4 (15 %) |  |
|  | **Other** | 2 (9 %) | 1 (4 %) |  |
| **Etiology, n (%)** | ***Surgery*** | 11 (48 %) | 13 (50 %) | 0.7564** |
|  | ***Acne*** | 7 (30 %) | 6 (23 %) |  |
|  | ***Trauma*** | 3 (13 %) | 3 (12 %) |  |
|  | ***Unknown*** | 1 (4 %) | 2 (8 %) |  |
|  | ***Vaccination*** | 0 (0 %) | 1 (4 %) |  |
|  | ***Burn injury*** | 0 (0 %) | 1 (4 %) |  |
|  | ***Piercing*** | 1 (4 %) | 0 (0 %) |  |
| **Previous treatments, n (%)** | ***None*** | 4 (17 %) | 12 (46 %) | 0.1955** |
|  | ***TAC*** | 17 (74 %) | 12 (46 %) |  |
|  | ***5-FU*** | 1 (4 %) | 1 (4 %) |  |
|  | ***Radiation and surgery*** | 1 (4 %) | 1 (4 %) |  |
| **Area (mm2)** | ***Median; 25 % and 75 % percentile*** | 978; 636, 1819 | 724; 444, 1596 | 0.1730*** |
|  | ***Range*** | 144–6899 | 169–3584 |  |
| *** T test, ** Chi square test, ***Mann-Whitney test** | | | | |

**Supplementary Table 2.** Area and cell density of different parts of keloid dermis before the treatment.

| **Dermis part** |  | **Area (mm²)** | **P-value** | **Cell density (pcs/mm²)** | **P-value** |
| --- | --- | --- | --- | --- | --- |
| Superficial dermis | Mean | 1 .03 | superficial dermis – deep dermis  P=0.0059 | 1465.43 | superficial dermis – deep dermis  P<0.0001 |
|  | SD; Range (5-95 %) | 0.46; 0.43–2.15 |  | 508.1; 7779.1 – 2382.0 |  |
| Middle dermis | Mean | 4. 81 | middle dermis – superficial dermis  P<0.0001 | 1588.75 | middle dermis – superficial dermis  P>0.9999 |
|  | SD; Range (5-95 %) | 2.15; 2.08–9.16 |  | 790.8; 516.9 – 3061.8 |  |
| Deep dermis | Mean | 1.42 | deep dermis – middle dermis  P<0.0001 | 2188.37 | deep dermis – middle dermis  P<0.001 |
|  | SD; Range (5-95 %) | 0.56; 0.37–2.39 |  | 685.0; 1010.4 – 3504.8 |  |

**Supplementary Table 3.** Myofibroblast density in different parts of keloid before the treatment in non-responders and responders.

|  | | | **No. (pcs/mm²)** | |  |
| --- | --- | --- | --- | --- | --- |
| **Dermis part** |  | | **Non-responders** | **Responders** | **P-value** |
| Superficial dermis | | Mean | 854.5 | 884.0 | P>0.9999 |
|  |  | SD; Range (5-95 %) | 240.6; 534.5–1388.8 | 352.2; 306.45–1803.7 |  |
| Middle dermis | | Mean | 1275.3 | 1332.0 | P>0.9999 |
|  |  | SD; Range (5-95 %) | 951.4; 371.4–3891.0 | 618.0; 484.9–2531.9 |  |
| Deep dermis | | Mean | 1303.2 | 1407.2 | P>0.9999 |
|  |  | SD; Range (5-95 %) | 612.9; 510.1–2932.9 | 600.8; 459.0–2860.8 |  |

**Supplementary Table 4.** The area of the keloid covered by myofibroblasts in keloids after the treatment. The change taking place in the myofibroblasts during the treatment is also expressed.

|  |  | **Non-responders** | |  | **Responders** | |  |
| --- | --- | --- | --- | --- | --- | --- | --- |
| **Dermis part** |  |  |  |  |  |  |  |
|  |  | **Post-treatment (%)** | **Change (pp)** | **P-value (change)** | **Post-treatment (%)** | **Change (pp)** | **P-value (change)** |
| Superficial dermis | Mean | 6.02 | 2.01 | P=0.2549 | 5.76 | 1.66 | P=0.7047 |
|  | SD; Range (5-95 %) | 4.63; 0.32–19.50 | 4.55; -7.01–14.82 |  | 4.94; 0.79–20.85 | 4.48; -3.03–15.08 |  |
| Middle dermis | Mean | 12.50 | 2.05 | P>0.9999 | 10.55 | -0.16 | P>0.9999 |
|  | SD; Range (5-95 %) | 11.15; 2.73–38.40 | 8.72; -10.97–28.71 |  | 11.18; 1.22–44.44 | 10.72; -14.02–32.13 |  |
| Deep dermis | Mean | 7.08 | -0.92 | P>0.9999 | 6.91 | - 1.59 | P=0.3623 |
|  | SD; Range (5-95 %) | 4.55; 1.58–17.68 | 6.94; -11.73–14.45 |  | 3.19; 3.03–13.52 | 3.39; -7.82–4.92 |  |

**Supplementary Table 5.** Percentage of myofibroblasts in keloids after the treatment. The change in the myofibroblast population that has taken place during the treatment is also given.

|  |  | **Non-responders** | |  | **Responders** | |  |
| --- | --- | --- | --- | --- | --- | --- | --- |
| **Dermis part** |  |  |  |  |  |  |  |
|  |  | **Post-treatment (%)** | **Change (pp)** | **P-value (change)** | **Post-treatment (%)** | **Change (pp)** | **P-value (change)** |
| Superficial dermis | Mean | 60.13 | 1.01 | P>0.9999 | 65.28 | 3.18 | P>0.9999 |
|  | SD; Range (5-95 %) | 14.00; 34.35–83.54 | 13.51; -25.79–31.43 |  | 13.19; 41.47–92.84 | 15.04; -21.09–29.23 |  |
| Middle dermis | Mean | 73.61 | -3.55 | P>0.9999 | 76.27 | -6.28 | P=0.2203 |
|  | SD; Range (5-95 %) | 18.51; 36.71–97.07 | 13.53; -36.12–16.32 |  | 10.76; 57.95–96.51 | 14.45; -30.91–23.45 |  |
| Deep dermis | Mean | 54.63 | -2.67 | P>0.9999 | 58.42 | -4.26 | P=0.6060 |
|  | SD; Range (5-95 %) | 8.77; 33.70–73.44 | 14.07; -28.46–16.05 |  | 11.48; 33.41–76.71 | 10.88; -20.92–17.98 |  |

**Supplementary Table 6.** Myofibroblast density in the keloids after the treatment and the change that has taken place during the treatment.

|  |  | **Non-responders** | |  | **Responders** | |  |
| --- | --- | --- | --- | --- | --- | --- | --- |
| **Dermis part** |  |  |  |  |  |  |  |
|  |  | **Post-treatment (pcs/mm²)** | **Change (pcs/mm²)** | **P-value (change)** | **Post-treatment (pcs/mm²)** | **Change (pcs/mm²)** | **P-value (change)** |
| Superficial dermis | Mean | 1116.2 | 261.7 | P=0.0188 | 1124.0 | 239.9 | P=0.0111 |
|  | SD; Range (5-95 %) | 488.7; 456.0–2170.5 | 399.0; -280.8–1002.8 |  | 440.1; 280.1–2044.0 | 384.1; -263.4–1240.2 |  |
| Middle dermis | Mean | 1399.2 | 123.8 | P>0.9999 | 1473.0 | 140.7 | P>0.9999 |
|  | SD; Range (5-95 %) | 999.1; 405.4–3923.7 | 707.4; -1044.6–2274.7 |  | 1164.0; 490.1–5051.0 | 1129.8; -1488.8–3000.8 |  |
| Deep dermis | Mean | 1029.6 | -282.4 | P=0.8145 | 1319.9 | -76.1 | P>0.9999 |
|  | SD; Range (5-95 %) | 521.1; 216.1–2531.1 | 654.9; -1803.7–665.8 |  | 657.1; 356.8–3113.5 | 599.8; -825.2–1456.0 |  |

**Supplementary Table 7.** Fraction and density of myofibroblasts in both treatment groups after the treatment.

|  |  | **Fraction of positive cells (%)** | |  | **Positive cell density (pcs/mm²)** | |  |
| --- | --- | --- | --- | --- | --- | --- | --- |
| **Dermis part** |  |  |  |  |  |  |  |
|  |  | **Non-responders** | **Responders** | **P-value** | **Non-responders** | **Responders** | **P-value** |
| Superficial dermis | Mean | 60.13 | 65.28 | P=0.7835 | 1116.2 | 1124.0 | P>0.9999 |
|  | SD; Range (5-95 %) | 14.00; 34.35–83.54 | 13.19; 41.47–92.84 |  | 488.7; 456.0–2170.5 | 440.1; 280.1–2044.0 |  |
| Middle dermis | Mean | 73.61 | 76.27 | P>0.9999 | 1399.2 | 1473.0 | P>0.9999 |
|  | SD; Range (5-95 %) | 18.51; 36.71–97.07 | 10.76; 57.95–96.51 |  | 999.1; 405.4–3923.7 | 1164.0; 490.1–5051.0 |  |
| Deep dermis | Mean | 54.63 | 58.42 | P>0.9999 | 1029.6 | 1319.9 | P=0.6672 |
|  | SD; Range (5-95 %) | 8.77; 33.70–73.44 | 11.48; 33.41–76.71 |  | 521.1; 216.1–2531.1 | 657.1; 356.8–3113.5 |  |

**Legends for Supplementary Figures**

**Supplementary Figure 1.** Modified CONSORT 2010 Flow Diagram for the RCT. Modified from Hietanen et al. “Treatment of keloid scars with intralesional triamcinolone and 5-fluorouracil injections – a randomized controlled trial” (22).

**Supplementary Figure 2.** Flow chart for the double-blinded RCT.
