# Supplementary figures and images for: Myofibroblasts reside in the middle dermis of the keloids but do not predict the response to injection therapies: a double-blinded, randomized, controlled trial
Source: Front Med (Lausanne). 2024 Mar 1;11:1293028. doi: 10.3389/fmed.2024.1293028 (PMC10943694; doi:10.3389/fmed.2024.1293028)

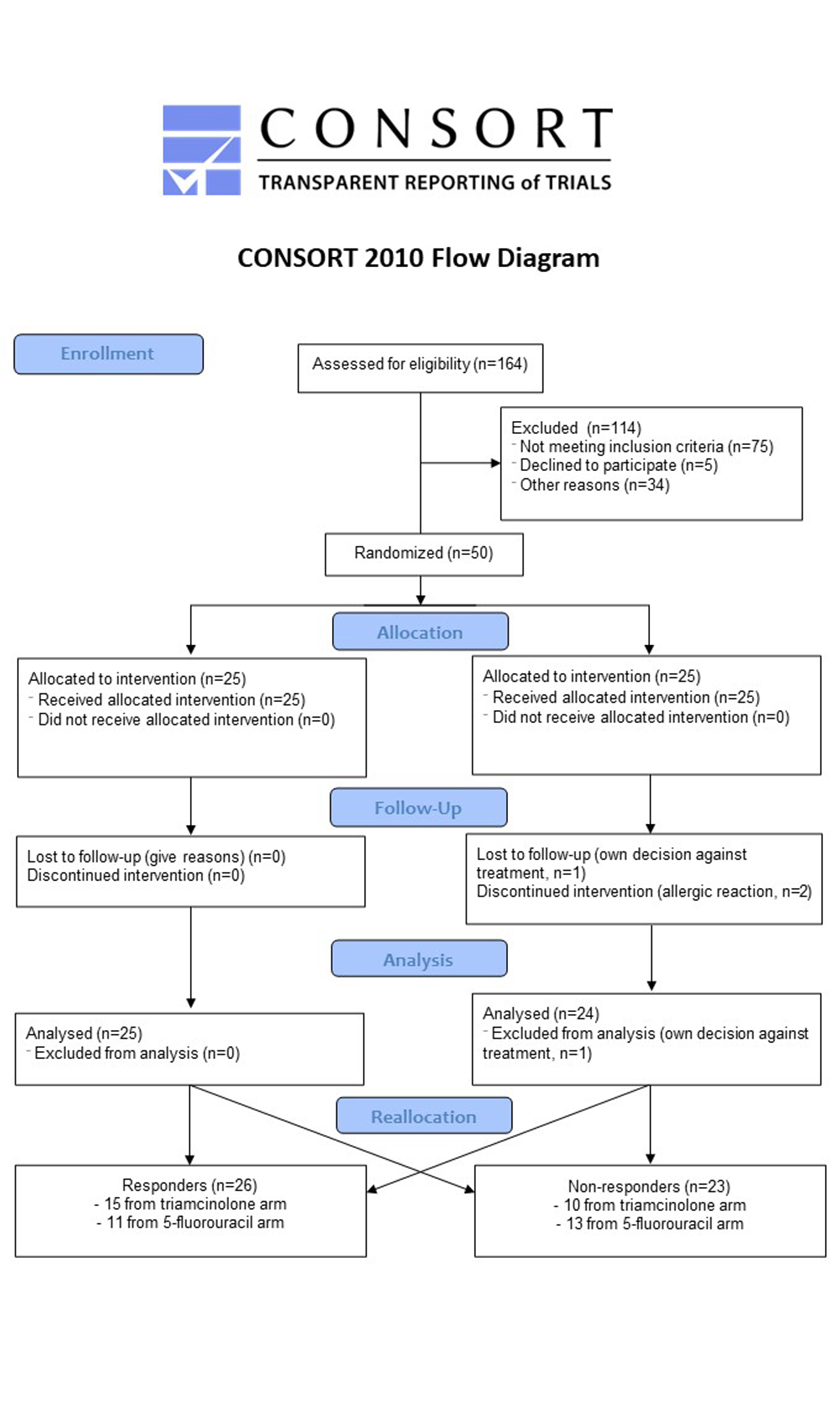

Supplement: Supplementary file 2 [file Image_1.JPEG]

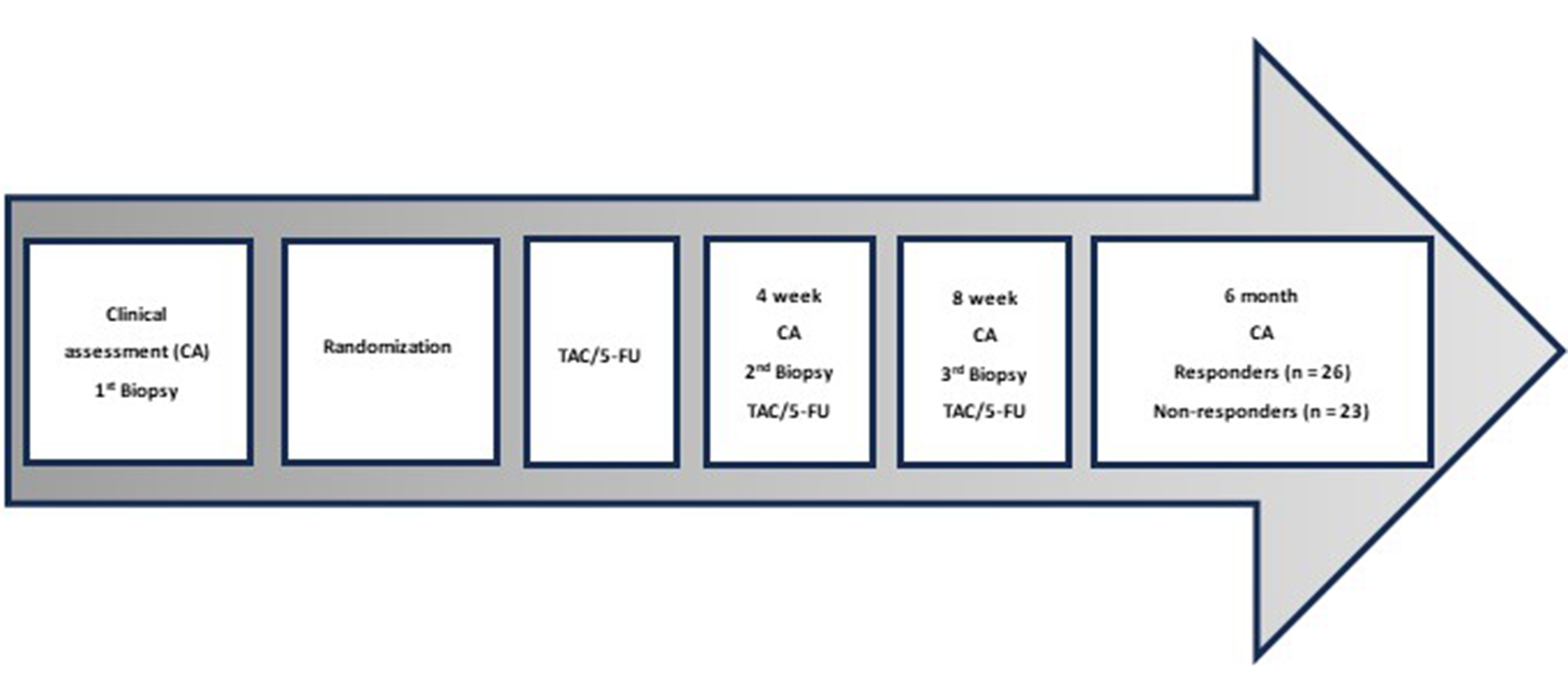

Supplement: Supplementary file 3 [file Image_2.JPEG]
